# Evaluating the Let's Move It intervention programme theory for adolescents' physical activity: Theorized psychosocial mechanisms of behavioural changes

**DOI:** 10.1111/bjhp.12744

**Published:** 2024-09-24

**Authors:** Minttu Palsola, Vera Araújo‐Soares, Wendy Hardeman, Ari Haukkala, Matti Toivo Juhani Heino, Falko Sniehotta, Reijo Sund, Tommi Vasankari, Nelli Hankonen

**Affiliations:** ^1^ Faculty of Social Sciences Tampere University Tampere Finland; ^2^ Faculty of Social Sciences University of Helsinki Helsinki Finland; ^3^ Division of Prevention, Center for Preventive Medicine and Digital Health (CPD), Medical Faculty Mannheim Heidelberg University Mannheim Germany; ^4^ University of Twente Enschede The Netherlands; ^5^ School of Health Sciences University of East Anglia Norwich UK; ^6^ Helsinki Collegium for Advanced Studies University of Helsinki Helsinki Finland; ^7^ NIHR Policy Research Unit Newcastle University Newcastle upon Tyne UK; ^8^ University of Eastern Finland Kuopio Finland; ^9^ UKK Institute for Health Promotion Research Tampere Finland; ^10^ Faculty of Medicine and Health Technology Tampere University Tampere Finland

**Keywords:** behaviour change technique enactment, intervention evaluation, physical activity, programme theory, randomized controlled trial, reasoned action approach, self‐determination theory

## Abstract

**Objectives:**

Behaviour change theories have extensively been used in health behaviour change interventions and their programme theories. However, they are rarely evaluated in randomized field studies. The Let's Move It intervention targeted various psychosocial constructs to increase adolescents' physical activity. A theory‐based process evaluation aiming to illuminate the trial findings as well as to test the programme theory used is conducted. Specifically, we investigate whether the intervention influenced the theorized determinants of change immediately post‐intervention and after 1 year, and whether these determinants were associated with changes in physical activity.

**Design:**

A cluster‐randomized controlled trial (*n* = 1166).

**Methods:**

We measured theorized determinants with self‐report, and physical activity (PA) with accelerometry and self‐report. The effects are evaluated with repeated measures ANOVA and regression models.

**Results:**

No changes were detected in most theorized determinants but intervention arm reported higher enactment of behaviour change techniques used during intervention immediately post‐intervention and lower descriptive norms for PA throughout. Autonomous motivation was associated with PA immediately post‐intervention.

**Conclusions:**

The lack of intervention effects may be due to many factors, for example insensitive measures, ceiling effects. However, reporting these null effects advances understanding of behaviour change processes. We introduce methodologic possibilities for future intervention programme theory evaluation efforts.

**Statement of contribution**

**What is already known on his subject?**

- School‐based physical activity interventions rarely assess interventions' mechanisms of behaviour change.
- School‐based physical activity interventions often have no or small effects on physical activity, which was also the case for the intervention under evaluation.
- Intervention evaluations enable active testing of theories or their core concepts, which help intervention developers evaluate what theories would be most useful for their purpose.

**What does this study add?**

- Use case of a state‐of‐the‐art approach to measure the mechanisms of behaviour change.
- Intervention arm students enacted behaviour change techniques taught in the intervention.
- Autonomous motivation was associated with increased PA.

## INTRODUCTION

How and why do behaviour change interventions work (or not)? To be able to answer this question, it is important to specify the rationale by which changes in behaviour are expected to occur (Davidoff et al., 2015; Skivington et al., 2021), that is lay out an intervention‐specific programme theory. Programme theories are practical and concrete working models that include assumptions about the mechanisms of behaviour change in terms of the intervention effects on the theorized determinants of behaviour (an action theory) and their relationship with the target behaviour (a conceptual theory; Chen, 1990). The key is to articulate the theories used in order to establish whether the intervention improved the target constructs, and if it did—how exactly (Davidoff et al., 2015)? This approach has long been advocated within physical activity (PA) intervention research (Baranowski et al., 1998). As behaviours such as PA are often complex, that is include multiple interacting components on different individual and environmental levels, and behavioural changes occur through various processes, it is justified for interventions to draw from multiple theories (Bartholomew Eldredge et al., 2016) to form intervention‐specific programme theories.

Outcome evaluations of interventions are common, but process evaluations are not as behavioural intervention studies rarely test the changes in these mechanisms alongside changes in target behaviours and outcomes (Hagger, Cameron, et al., 2020). This is also true for school‐based interventions among older adolescents aged 15–19 years (Hynynen et al., 2016). To better understand how and why an intervention works or not, efforts should also be guided towards evaluating the theoretical assumptions about the mechanisms of change, that is the behaviour change processes that the underlying theories assume to be induced by the intervention (Hagger, Moyers, et al., 2020). These evaluations of behaviour change processes provide valuable information on the feasibility of the underpinning theories of interventions and advance theory development (see also Rothman, 2004). This is crucial since theories provide frameworks to identify potentially effective intervention content and the mechanisms of change (Hagger, Moyers, et al., 2020; Hankonen & Hardeman, 2020). Intervention evaluations can lead to a virtuous cycle that enables active testing of, if not a theory, at least its core concepts, which help intervention developers evaluate what theories would be most useful for their purpose (Davidoff et al., 2015). To advance the science of behaviour change, it is essential to conduct theory‐based process evaluations.

Despite rich literature on school‐based physical activity (PA) interventions among children and young adolescents, only few methodologically high‐quality field trials have been conducted among older adolescents (Hynynen et al., 2016). Even fewer trials focus on older adolescents (aged 15–20 years) in vocational education settings (Grüne et al., 2020), despite inadequate PA being a prevalent health risk among those with lower education (Elgar et al., 2015). Few school‐based PA interventions have demonstrated effects on accelerometry‐measured activity (Love et al., 2019), or achieved significant changes in their theorized behavioural determinants, that is factors that affect behaviours, for PA change (van Stralen et al., 2011). To our knowledge, there has not been any conclusive research on the mechanisms of change of school‐based interventions in the more recent years. Regarding conceptual theory, many hypothesized mechanisms of intervention effects on PA among school‐age children and adolescents aged 4 to 18 years exist (e.g. Lubans et al., 2008; van Stralen et al., 2011). Studies have identified, for example self‐efficacy, outcome expectancy, intention, self‐regulation, intrinsic motivation and autonomy support as significant determinants for school‐age children and adolescents (Kelso et al., 2020; Lubans et al., 2008; van Stralen et al., 2011). Among upper‐secondary school students, for example PA identity (e.g. to what extent being physically active is (not) aligned with one's self‐concept), intention and self‐monitoring have been identified as potential determinants (Hankonen et al., 2017).

**FIGURE 1:**
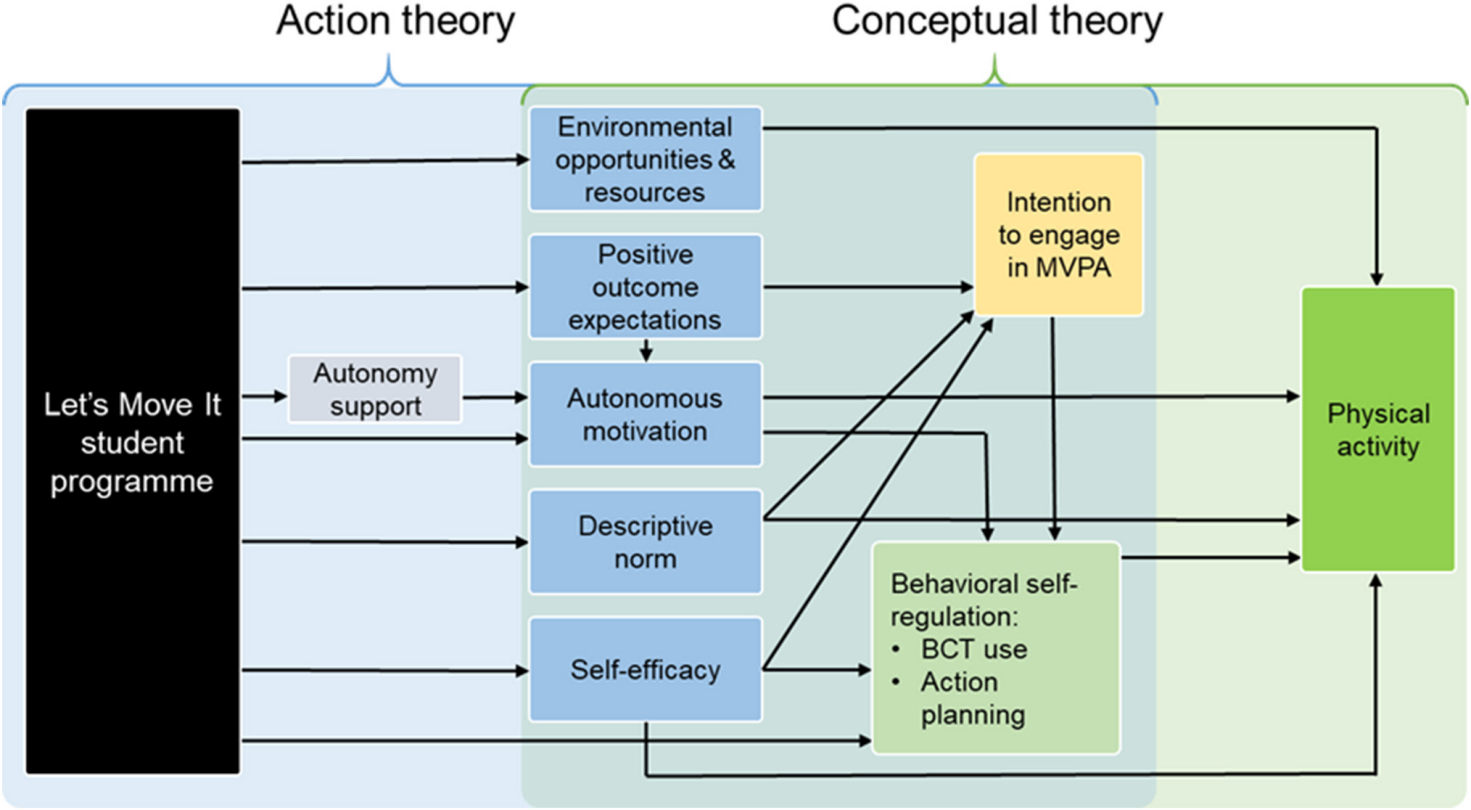
Visualization of the mechanisms of change set by the programme theory under evaluation. The intervention programme theory draws on several formal theories and is a combination of those. Action theory refers to the assumed links between the programme components and the theoretical determinants, whereas conceptual theory refers to the assumed links between theoretical determinants and the target behaviour (PA).

This study aimed to fill these gaps by conducting a theory‐based process evaluation which aims to both illuminate the trial findings of Let's Move It (LMI), as well as test programme theory of vocational school students' PA change (Hankonen et al., 2020) in a cluster‐randomized controlled trial (Hankonen et al., 2016). The focus is on the assumptions posed by the LMI programme theory (see Figure 1) and the underlying social psychological theories of behaviour change (reasoned action approach; RAA, self‐determination theory; SDT, control theory) about the mechanisms of change (see Hankonen et al., 2020 for more details). In particular, this paper assesses mechanisms of impact because the intervention to target vocational school students' PA was developed based on particular theories which have not extensively been tested. We also investigated fidelity of implementation elsewhere (see Hankonen et al., 2023).

The intervention aimed at increasing moderate‐to‐vigorous physical activity (MVPA), that is activities that produce big increases in heart rate and breathing such as brisk walking or biking, among those with low/moderate baseline levels and at decreasing sedentary behaviour among all participants, but the analyses of the main intervention effects (reported in Hankonen et al., 2023) revealed important and statistically significant positive effects only for sedentary behaviours post‐intervention. This paper examines explanations for not finding effects on MVPA in the outcome evaluation (Hankonen et al., 2023). Random variation due to lack of power, measurement error and other sources of uncertainty may play an important role, but there are alternative explanations in relation to the theorized mechanisms of change, both in the action and conceptual theory, which we investigate here. First, as the intervention was not (demonstrated to be) effective, it is possible that the intervention might have not been effective in changing the theorized determinants of MVPA change (action theory). Second, the intervention may have affected the determinants, but the determinants are not powerful in changing MVPA in the trial population, that is the conceptual theory behind LMI did not work as assumed (conceptual theory).

## OBJECTIVES

We investigated whether detecting no changes in MVPA could be attributed to (1) the intervention not having an effect on the theorized determinants of change (action theory) or (2) the determinants not being associated with behaviour change in the trial population (conceptual theory). The research questions (RQs) were as follows:

RQ1: Did the Let's Move It intervention influence the psychosocial theorized determinants (as assumed by the action theory), immediately post‐intervention (T3) and were any changes detectable at 1‐year follow‐up (T4)?
RQ2: Are the theorized determinants associated with (changes in) PA?

The overarching hypothesis of the programme theory was that the intervention would induce positive changes in the theorized mechanisms of change for PA. The research questions are based on pre‐registered hypotheses (https://osf.io/h2uaq/).

## METHODS

### Study setting

LMI intervention was evaluated in a cluster‐randomized controlled trial (trial registration: ISRCTN10979479) in six vocational schools in Finland. We use study measurements conducted at baseline (T1), and two (T3) and 12–14 months after baseline (T4) (mid‐intervention measurement, T2, excluded as it contains only the intervention arm; details in Hankonen et al. (2016)). The ethical committee of the Hospital District of Helsinki and Uusimaa (367/13/03/03/2014) reviewed the procedures. Informed consent was obtained from all participants included in the study.

This paper examines the assumptions behind the student programme targeting MVPA (see Köykkä et al., 2019 for teacher programme). The student programme included six weekly group sessions over 2 months and a booster session, a poster campaign, active classrooms, and PA opportunity enhancement both in and outside school settings by modified choice architecture (for details, see Hankonen et al., 2016, 2020). The theorized mechanisms of change are part of the intervention programme theory that guided the intervention facilitators' activities, materials and workbook designs, etc. (Supplementary File 1). The components, theorized behavioural determinants and causal assumptions were based on RAA (outcome expectancies, perceived behavioural control, descriptive norm, intention and its social‐cognitive antecedents; Fishbein & Ajzen, 2011), SDT (autonomous motivation and internalization of motivation; Deci & Ryan, 2000) and control theory (self‐regulation; Carver & Scheier, 1982). The focus is on investigating the assumptions set by the action and conceptual theory, informed by formal theories.

### Sample

At baseline, out of 1166 participants, 638 were in the intervention arm (see Table 1 in Heino et al., 2019 for details on baseline sample demographics). The participants were aged 15–49 (full sample Mean = 17.95, Mdn = 17.00 SD = 3.4; intervention arm *N* = 590, Mean = 18.18, Mdn = 17.00, SD = 3.76; control arm *N* = 514, Mean = 17.68, SD = 2.92). The variation in age is not uncommon in the Finnish vocational school system which is designed for both young people finishing their basic schooling as well as for adults already in work life. Those who self‐rated having less than quite good Finnish proficiency (comprehension and oral; *N* = 41) were excluded. Analyses were performed separately for those with low‐to‐moderate baseline PA levels, excluding the most active 20% of the sample. These exclusions were pre‐defined in the trial protocol (Hankonen et al., 2016).

### Measures

Moderate‐to‐vigorous physical activity was measured with accelerometry and self‐report (Heino et al., 2019), as they measure different aspects of MVPA: self‐reports inquire about frequency, whereas accelerometry also assesses total volume and avoids reporting biases. In self‐reports, participants indicated the number of days during the past week (from 0 to 7) they engaged in more than 30 min of MVPA (NordPAQ measure; Rasmussen, 2012). MVPA was described to the respondents in lay terms as ‘FREE TIME physical activity that increases your heart rate and makes you catch your breath and in which you engage at least for 1.5 h a week. The weekly amount can accumulate in various ways, for example from three separate half‐an‐hour sessions, or from six separate 15‐min sessions, or from two 45‐min physical activity sessions. Physical activity that increases your heart rate and makes you catch your breath includes for example brisk walking, biking to school, ball games, running, skateboarding, snowboarding, dancing, gym training or group training’. Objective PA was measured with a hip‐worn tri‐axial accelerometer (Hookie AM 20, Traxmeet Ltd, Espoo, Finland) and by analysing raw acceleration data with a mean amplitude deviation (MAD) algorithm (Vähä‐Ypyä, Vasankari, Husu, Suni, et al., 2015). Analyses using MAD algorithms are based on raw acceleration data, and no data processing or cleaning was used (details concerning analyses are presented in Vähä‐Ypyä, Vasankari, Husu, Mänttäri, et al., 2015; Vähä‐Ypyä, Vasankari, Husu, Suni, et al., 2015). The cut point between light and moderate PA was set to 3.0 metabolic equivalent using MAD cut‐point value 91 milligravity (based on hip‐worn data for adults; Vähä‐Ypyä, Vasankari, Husu, Mänttäri, et al., 2015). The participants were instructed to wear the accelerometer fixed on an elastic belt on their hip for seven consecutive days during waking hours, except during showering and other water activities. Accelerometers were handed to participants personally at schools by research assistants upon instructing the correct usage. For returning the accelerometers, mailboxes were set up at the schools, and for the students who did not return their accelerometer in the mailbox, a postage‐paid return envelope was sent to their home address. PA analyses focused on the time the participants wore the accelerometer. The data inclusion criterion was 4 days of at least 10 h of data, including at least one weekend day. This protocol is similar to the majority of the studies using accelerometer‐based PA as well as the one used in population‐based samples of Finnish children and adolescents (Jussila et al., 2022) and adults (Husu et al., 2016).

Mean scores were used for all sum variables of the self‐reported constructs. Unless referenced otherwise, the measures have specifically been adapted for this study based on the guidance by Francis et al. (2004). Positive outcome expectations were measured with 12 items on a scale from 1 = completely disagree to 7 = completely agree (‘What kind of consequences do you expect there to be, if you were physically active weekly at least 1.5 h in a way that increases your heart rate and makes you catch your breath?’) for example ‘It would put me in a good mood’ (at baseline, *α* = .918). Descriptive norms were measured with two items on a scale from 1 = not at all true to 7 = completely true, by asking if respondents' friends and parents were regularly physically active at least 1.5 h a week (*α* = .499). Intention was measured with one item on the likelihood (1 = unlikely, 7 = likely) and one on certainty (1 = definitely not, 7 = definitely yes) of engaging in at least 1.5 h of PA a week during the next month (*α* = .941). The distribution was skewed (Mdn = 6.00, Q_1_ = 4.00, Q_3_ = 7.00). Self‐efficacy was measured on a scale from 1 = strongly disagree to 7 = strongly agree, for example ‘If I wanted to, I could be regularly physically active’, as was perceived behavioural control, for example ‘I have full control over whether or not to be regularly physically active’. Altogether, there were five items (*α* = .607). Autonomous motivation was measured with nine items on a scale from 1 = not at all true to 7 = completely true, for example ‘I am physically active because it is fun’ (*α* = .941) (Markland & Tobin, 2004; Wilson et al., 2006). Environmental opportunities were measured with eight items at baseline and seven at follow‐ups on a scale from 1 = not at all true to 7 = completely true, for example ‘I have enough money to be physically active’ (*α* = .629). Behaviour change technique (BCT; Michie et al., 2013) scales were fully developed for this study, measuring BCT use either by identification of use (10 items; 1 = not at all true, 6 = completely true; *α* = .915) or frequency of use (eight items; 1 = not once, 2 = once, 3 = twice, 4 = weekly, 5 = about every second day, 6 = daily), for example ‘I have planned for ways to overcome barriers for doing PA’ (*α* = .866). Action and coping planning were measured with four items for each on a scale from 1 = completely disagree to 4 = completely agree, for example ‘I have made a detailed plan regarding how often to exercise’ (action planning *α* = .938; coping planning *α* = .910) (Sniehotta et al., 2005). For a more comprehensive description of the baseline measures, see the supplementary website https://git.io/fAj0e for Heino et al. (2019) and the study protocol (Hankonen et al., 2016).

### Statistical analyses

Alpha was set at 5% for all analyses. For investigating whether the intervention had affected the theorized determinants (RQ1), repeated measures ANOVAs were conducted, first from baseline to post‐intervention, and then across all measurement points. Baseline values of age and gender, educational track (4 in total) and parental socioeconomic status based on their education level (basic education, mid‐level education, higher education, unknown) and countries of birth (both born in Finland, other) were controlled for in the models, due to indications of between‐arm differences and significant relationships with accelerometry‐PA at baseline (Hankonen et al., 2023; Heino et al., 2019). The control variables were accounted for in separate models with one control variable for each (six models per determinant). Due to multiple testing, Type 1 error rate was controlled for and Bonferroni corrections (Bland & Altman, 1995) were used when making claims about the action theory. For each theorized determinant, the *p* values were corrected for six tests. Wherever a statistically significant effect for the intervention was detected, changes in estimated marginal means were investigated. Due to violations of sphericity (Mauchly's test; *p* < .05) and *p* > 0.75 for all theorized determinants, Huynh‐Feldt adjusted values are reported (Field, 2009; Tabachnick & Fidell, 2014). Tests of tolerance (lowest value 0.296) and VIF (highest value 3.377) indicated no issues with multicollinearity. We follow the same analysis strategy as in the evaluation of the sitting reduction component of the intervention (Aulbach et al., 2023).

The associations between the theorized determinants and MVPA at post‐intervention and follow‐up (RQ2) were investigated with correlation analyses and nested linear multivariable regression models. Prior to the regression analysis, bivariate correlations between theorized mechanisms of changes in PA, and accelerometry and self‐reported PA were investigated for the whole cohort. As action and coping planning were also included in the BCT measures, including them in the regression model might over‐emphasize their effects. Due to this and high correlations, action and coping planning were excluded from the regression models. The order of the included (groups of) variables was decided following the order of the programme theory. In Step 1, the first‐order theorized determinants were included. In Step 2, intention and behavioural regulation. In the final step, the model was adjusted for baseline levels of MVPA to examine the changes in MVPA. No major deviations from the assumptions of linearity, homoscedasticity or independence were detected when inspecting residual scatter plots, Mahalanobis and Cook's distances and Durbin‐Watson statistics. Two‐tailed tests were used. IBM Statistics SPSS version 25.0 was used. All analyses were repeated with and without the active participants.

## RESULTS

### RQ 1: Did the Let's Move It intervention influence the psychosocial theorized determinants (action theory)?

#### From baseline to immediate post‐intervention

The analyses indicate few intervention effects on the theorized determinants (Table 1). Between‐group difference was detected in BCT use: it statistically significantly increased in the intervention arm, whereas there were no changes in the control arm. This difference in BCT use persisted when controlling for gender *F*(1, 829) = 8.400, eta^2^ = 0.010, *p* = .024 and educational track *F*(1, 825) = 7.870, eta^2^ = 0.009, *p* = .030.

**TABLE 1:** Intervention effects on theorized determinants from baseline (T1) to immediately post‐intervention (T3).

Measure	Intervention (*n* = 438^a^)		Control (*n* = 395^b^)		*F*	*η* ^2^
	*M* (SD) T1	*M* (SD) T3	*M* (SD) T1	*M* (SD) T3		
1. Positive outcome expectations	5.24 (1.24)	5.26 (1.34)	5.26 (1.25)	5.35 (1.14)	*F*(1, 835)	
Time					1.650	0.002
Time × Arm					0.414	0.001
2. Descriptive norm	4.41 (1.58)	4.20 (1.58)	4.55 (1.54)	4.52 (1.56)	*F*(1, 836)	
Time					5.562	0.007
Time × Arm					3.685	0.004
3. Intention	5.48 (1.80)	5.11 (1.88)	5.33 (1.91)	5.20 (1.84)	*F*(1, 834)	
Time					19.691	0.023***
Time × Arm					4.200	0.005
4. Self‐efficacy/perceived behavioural control	5.32 (1.09)	4.92 (1.07)	5.33 (1.07)	5.06 (1.07)	*F*(1, 837)	
Time					84.773	0.092***
Time × Arm					3.431	0.004
5. Autonomous motivation	3.41 (1.04)	3.44 (1.05)	3.46 (1.06)	3.49 (1.05)	*F*(1, 834)	
Time					1.377	0.002
Time × Arm					0.018	0.000
6. Environmental opportunities and resources	4.98 (1.02)	4.88 (1.07)	5.00 (0.98)	4.96 (0.94)	*F*(1, 838)	
Time					4.930	0.006
Time × Arm					0.986	0.001
7. BCT use (identification of use)	3.02 (1.33)	3.14 (1.33)	3.21 (1.39)	3.15 (1.38)	*F*(1, 833)	
Time					5.307	0.006
Time × Arm					4.515	0.005
8. BCT use (frequency of use)	2.44 (1.11)	2.51 (1.16)	2.71 (1.26)	2.55 (1.18)	*F*(1, 831)	
Time					16.468	0.019***
Time × Arm					8.184	0.010*
9. Action planning	2.77 (0.90)	2.74 (0.95)	2.70 (0.94)	2.70 (0.94)	*F*(1, 834)	
Time					2.845	0.003
Time × Arm					0.326	0.000
10. Coping planning	2.49 (0.87)	2.49 (0.88)	2.44 (0.93)	2.48 (0.92)	*F*(1, 834)	
Time					1.298	0.002
Time × Arm					0.262	0.000

*Note*: *p < .05; ***p < .001 (Bonferroni corrected *p* values to account for controlled analyses 6 tests in total per determinant).

^a^ Varies between 438 and 443.

^b^ Varies between 395 and 396.

#### From baseline until 14‐month follow‐up

Over‐time changes in both trial arms were mostly similar (Table 2). However, the intervention arm's scored lower in descriptive norms than the control arm across time points (eta^2^ = 0.015, *p* = .027), but there were no between‐group differences in the change patterns across the time points (eta^2^ = 0.002, *p* = .899). This pattern in descriptive norms was detectable when controlled for gender (*F*(1, 544) = 7.787, eta^2^ = 0.014, *p* = .030), but not with other control variables.

**TABLE 2:** Intervention effects on theorized determinants from baseline (T1) via post‐intervention (T3) to follow‐up (T4).

Measure	Intervention (*n* = 254^a^)			Control (*n* = 288^b^)			*F* ^c^	*η* ^2^
	*M* (SD) T1	*M* (SD) T3	*M* (SD) T4	*M* (SD) T1	*M* (SD) T3	*M* (SD) T4		
1. Positive outcome expectations	5.14 (1.29)	5.30 (1.31)	5.29 (1.32)	5.22 (1.21)	5.30 (1.13)	5.33 (1.21)	*F*(1.95, 1072.61)	
Time							3.818	0.007
Time × Arm							0.270	0.000
2. Descriptive norms	4.36 (1.51)	4.19 (1.55)	4.16 (1.61)	4.60 (1.56)	4.61 (1.51)	4.43 (1.53)	*F*(1.98, 1078.87)	
Time							4.298	0.008
Time × Arm							1.147	0.002
3. Intention	5.33 (1.86)	5.04 (1.94)	5.13 (1,82)	5.38 (1.89)	5.24 (1.80)	5.16 (1.90)	*F*(1.97, 1073.89)	
Time							5.405	0.010*
Time × Arm							0.766	0.001
4. Self‐efficacy/perceived behavioural control	5.30 (1.03)	4.96 (1.04)	5.12 (0.99)	5.36 (1.05)	5.08 (1.03)	5.18 (1.09)	*F*(1.96, 1070.70)	
Time							22.960	0.040***
Time × Arm							0.264	0.000
5. Autonomous motivation	3.36 (1.07)	3.48 (1.07)	3.45 (1.03)	3.45 (1.06)	3.48 (1.04)	3.42 (1.09)	*F*(1.92, 1050.14)	
Time							2.563	0.005
Time × Arm							1.723	0.003
6. Environmental opportunities and resources	5.07 (0.99)	5.00 (1.03)	5.06 (0.96)	5.07 (0.97)	5.01 (0.92)	5.07 (1.05)	*F*(1.96, 1073.96)	
Time							1.673	0.003
Time × Arm							0.030	0.000
7. BCT use (identification of use)	2.97 (1.30)	3.22 (1.40)	2.88 (1.38)	3.18 (1.32)	3.18 (1.39)	3.08 (1.36)	*F*(1.95, 1063.30)	
Time							8.032	0.015**
Time × Arm							2.788	0.006
8. BCT use (frequency of use)	2.35 (1.06)	2.65 (1.24)	2.46 (1.22)	2.51 (1.14)	2.58 (1.20)	2.50 (1.20)	*F*(1.98, 1071.12)	
Time							6.777	0.012**
Time × Arm							2.579	0.005
9. Action planning	2.71 (0.91)	2.74 (0.96)	2.57 (0.98)	2.78 (0.92)	2.71 (0.94)	2.70 (0.99)	*F*(1.97, 1071.51)	
Time							4.474	0.008
Time × Arm							2.060	0.004
10. Coping planning	2.47 (0.87)	2.47 (0.95)	2.35 (0.89)	2.56 (0.88)	2.47 (0.92)	2.47 (0.97)	*F*(1.98, 1081.09)	
Time							3.778	0.007
Time × Arm							1.311	0.002

*Note*: For descriptive norms, a main effect for arm was detected *F*(1, 546) = 8.149, *η* ^2^ = 0.015, *p* = .027. *p < .05; **p < .01; ***p < .001 (Bonferroni corrected *p* values to account for controlled analyses 6 tests in total per determinant).

^a^ Varies between 254 and 257.

^b^ Varies between 288 and 293.

^c^ Huynh–Feldt adjusted degrees of freedom reported due to indications of sphericity violations in Mauchly's test.

#### Changes among the low‐to‐moderately active participants

No significant between‐arm differences were found in the change patterns from baseline neither to immediately post‐intervention nor 14 months.

### RQ2: Are the theorized determinants associated with (changes in) PA (conceptual theory)?

Most theorized determinants were related to PA cross‐sectionally at baseline (see Supplementary File 2): mostly small positive significant correlations with accelerometry MVPA (0.11 ≤ *r* ≤ 0.25, *p* < .001), small to medium correlations with self‐reported MVPA (0.19 ≤ *r* ≤ 0.42, *p* < .001), all in the expected direction. Opportunities, outcome expectations and descriptive norms were not associated with accelerometry MVPA. The size and significance of the correlation coefficients were similar at all measurement points. Post‐intervention, of all the theorized determinants, autonomous motivation indicated the highest bivariate correlation with MVPA measures (*r* = .25, *p* < .001 with accelerometry MVPA, and *r* = .41, *p* < .001 with self‐reported MVPA).

Post‐intervention values of the theorized determinants were used as independent variables in the stepwise linear regression models. The highest detected correlations at post‐intervention were between autonomous motivation and intention (*r* = .64), identification of use BCTs (*r* = .52, *p* < .001), action planning (*r* = .55, *p* < .001) and coping planning (*r* = .62, *p* < .001).

At post‐intervention (Table 3), autonomous motivation was positively associated with accelerometry MVPA levels, and this association persisted even when baseline behaviour was controlled for. At 14 months (Table 4), only autonomous motivation was associated with accelerometry MVPA levels, but this association did not persist when controlling for baseline behaviour. The negative associations of positive outcome expectations and environmental opportunities at 14 months are most likely statistical artefacts (due to e.g. multicollinearity or conditioning on a collider; Elwert & Winship, 2014) as bivariate associations were zero or positive.

**TABLE 3:** Stepwise regression of the associations between theorized determinants for PA change and accelerometry MVPA at post‐intervention.

Effect	Estimate	SE	95% CI		*p*
			*LL*	*UL*	
Step 1					
(Constant)	57.052	8.574	40.197	73.907	<.001
Positive outcome expectations	−3.531	1.514	−6.508	−0.554	.020
Descriptive norms	−.659	.962	−2.551	1.232	.494
Autonomous motivation	11.047	1.798	7.512	14.581	<.001
Environmental and physical opportunities	−3.864	1.650	−7.106	−0.621	.020
Self‐efficacy	2.030	1.516	−0.951	5.011	.181
Step 2					
(Constant)	57.895	8.794	40.608	75.183	<.001
Positive outcome expectations	−3.625	1.526	−6.625	−0.626	.018
Descriptive norms	−.916	.995	−2.871	1.039	.358
Autonomous motivation	9.915	2.241	5.509	14.321	<.001
Environmental opportunities	−3.781	1.674	−7.072	−0.489	.024
Self‐efficacy	1.816	1.542	−1.216	4.847	.240
Intention to engage in PA	.781	1.090	−1.362	2.924	.474
BCT use (identification)	1.453	1.614	−1.720	4.625	.369
BCT use (frequency)	−1.231	1.746	−4.663	2.200	.481
Step 3					
(Constant)	21.800	7.173	7.699	35.900	.003
Positive outcome expectations	−1.501	1.192	−3.843	0.842	.209
Descriptive norms	−0.271	0.773	−1.791	1.249	.726
Autonomous motivation	4.077	1.776	0.585	7.569	.022
Environmental and physical opportunities	−2.149	1.304	−4.712	0.414	.100
Self‐efficacy	0.708	1.199	−1.649	3.066	.555
Intention to engage in PA	1.263	.847	−0.402	2.928	.137
BCT use (identification)	−0.123	1.257	−2.593	2.348	.922
BCT use (frequency)	−0.989	1.355	−3.653	1.676	.466
Baseline MVPA	0.627	0.038	0.552	0.702	<.001

*Note*: *N* = 416, *R* ^2^ = .10, *R* ^2^ adj. = .09 for Step 1, *R* ^2^ = .11, *R* ^2^ adj. = .09 for Step 2, *R* ^2^ = .46, *R* ^2^ adj. = .45 for Step 3. Δ*R* ^2^ = .10 for Step 1 (*p* < .001), Δ*R* ^2^ = .00 for step 2 (*p* = .658), Δ*R* ^2^ = .36 for step 3 (*p* < .001).

Abbreviations: CI, confidence interval; *LL*, lower limit; *UL*, upper limit.

**TABLE 4:** Stepwise regression model of the associations between theorized determinants for PA change and accelerometry MVPA at 14‐month follow‐up.

Effect	Estimate	SE	95% CI		*p*
			*LL*	*UL*	
Step 1					
(Constant)	38.586	9.239	20.407	56.764	<.001
Positive outcome expectations	−.981	1.636	−4.200	2.238	.549
Descriptive norms	.999	1.033	−1.034	3.032	.334
Autonomous motivation	7.106	1.940	3.289	10.924	<.001
Environmental opportunities	−1.420	1.771	−4.905	2.064	.423
Self‐efficacy	.889	1.639	−2.337	4.115	0.588
Step 2					
(Constant)	38.782	9.507	20.075	57.488	<.001
Positive outcome expectations	−.992	1.654	−4.247	2.262	.549
Descriptive norms	.937	1.071	−1.171	3.045	.383
Autonomous motivation	6.850	2.443	2.043	11.657	.005
Environmental and physical opportunities	−1.439	1.802	−4.985	2.107	.425
Self‐efficacy	.812	1.668	−2.470	4.095	.627
Intention to engage in PA	.512	1.186	−1.822	2.846	.666
BCT use (identification)	−.577	1.752	−4.024	2.870	.742
BCT use (frequency)	.263	1.885	−3.446	3.972	.889
Step 3					
(Constant)	13.128	8.717	−4.024	30.280	.133
Positive outcome expectations	0.428	1.454	−2.434	3.289	.769
Descriptive norms	1.412	0.938	−0.434	3.259	.133
Autonomous motivation	2.521	2.182	−1.773	6.815	.249
Environmental and physical opportunities	−0.223	1.581	−3.334	2.889	.888
Self‐efficacy	−0.003	1.462	−2.879	2.873	.998
Intention to engage in PA	0.888	1.038	−1.155	2.931	.393
BCT use (identification)	−1.585	1.536	−4.607	1.437	.303
BCT use (frequency)	0.344	1.649	−2.900	3.588	.835
Baseline MVPA	0.456	0.046	0.364	0.547	<.001

*Note*: *N* = 319, *R* ^2^ = .07, *R* ^2^ adj. = .06 for Step 1, *R* ^2^ = .07, *R* ^2^ adj. = .05 for Step 2, *R* ^2^ = .29, *R* ^2^ adj. = .48 for Step 3. Δ*R* ^2^ = .07 for Step 1 (*p* < .001), Δ*R* ^2^ = .00 for step 2 (*p* = .968), Δ*R* ^2^ = .22 for step 3 (*p* < .001).

Abbreviations: CI, confidence interval; *LL*, lower limit; *UL*, upper limit.

## DISCUSSION

We set out to investigate whether the reason why the LMI intervention did not detectably increase MVPA (Hankonen et al., 2023) was attributable to (1) the intervention not being able to change theorized determinants (action theory), or (2) these constructs not being associated with the target behaviour in this population (conceptual theory). We found no effect of the intervention on the theorized determinants at post‐intervention or 14‐month follow‐up, except for BCT use at post‐intervention. Support was found for parts of the conceptual theory, as most of the theorized determinants significantly correlated with accelerometry‐measured and self‐reported PA in the expected direction. Autonomous motivation was associated with levels and changes in accelerometry‐measured MVPA immediately post‐intervention as expected.

Compared with previous literature, not detecting intervention effects on the theorized determinants has also been the case for several other school‐based PA interventions (van Stralen et al., 2011). Reporting these null effects advances our understanding of intervention processes and effects as they can keep us from repeating the same errors. These results complement qualitative process evaluation findings, drawing on semi‐structured interviews among a subsample (Kostamo et al., 2019; Palsola et al., 2020; Renko et al., 2022). The interviews indicated that the participants perceived the BCTs useful, but they also reported struggling with integrating the content and BCTs into their daily lives outside the intervention setting (Palsola et al., 2020). Hence, some of the increase in reported BCT use detected here might have occurred during the intervention activities. In terms of the planning BCTs, the participants have voiced many reasons not to make plans, mostly related to anticipated negative feelings as a consequence of planning, for example PA might start to feel forced or one might be annoyed in case the plan fails (Renko et al., 2022). The qualitative findings also indicated that the intervention was well received and LMI content was perceived as a source of motivation for PA (Kostamo et al., 2019; Palsola et al., 2020), but here we detected no changes in motivation on average.

### Explanations for the findings

Next, we discuss potential explanations for why this study did not find significant increases in most theorized mechanisms. As stated earlier, there are many sources of uncertainty that cannot be ruled out based on the current study.

In relation to the measures, the participants may have perceived the target PA level defined in the survey too easy, resulting in negatively skewed theorized determinant variables, leading to ceiling effects. All items were related to achieving a target of 90 min of MVPA in any increments throughout the week, with daily activities counting towards the target: Participants may have seen this easily achievable and given high ratings on the items already at baseline (Heino et al., 2019). These ceiling effects (i.e. no room for improvement) lead to low predictive power. Not detecting changes in previous adolescent PA research has also been attributed to ceiling effects (Smith et al., 2018). Also, measures of descriptive norms, self‐efficacy and environmental opportunities had low alphas, indicating that the questions included in these sumscores might measure slightly different things. Insensitive measures have been problematic in other school‐based PA intervention studies, too (van Stralen et al., 2011). Furthermore, ‘the initial elevation bias’, an issue in repeated assessments, refers to apparent decreases over‐time in survey answers that do not reflect actual drop (Shrout et al., 2018). Therefore, there may have been actual changes in cognitions, as indicated by qualitative evaluations.

In terms of the intervention programme itself, the fidelity may have been too low or the intervention dose too small (see e.g. Oldenburg et al., 2010 for evidence that more intensive and longer interventions are more effective). Here, low *delivery* fidelity is not likely, as according to facilitator self‐reports 97.3% of the intended activities were delivered (Hankonen et al., 2023). However, problems might have arisen on the *participant level* as the programme was designed for older adolescents (aged 15–19), but almost one fifth of the sample was older. It is possible that the programme was not received as intended among the older participants, leading to non‐detectable changes in social cognitions.

In terms of the target population, it was particularly challenging: In Finland, children from lower socioeconomic status families are more likely to attend vocational education track than the more academic track. Of the notable differences between the higher and lower socioeconomic status students' PA, the reasons may at least partly lie in factors, for example higher levels of stress related to economic situation (Hankonen et al., 2017), outside the scope of this intervention. In the presence of these external stressors, the adolescents might not have the resources or energy for behaviour change efforts. Based on a qualitative evaluation of the participants' engagement with the intervention, they did indeed understand the content but struggled with carrying out the BCTs (e.g. planning and self‐monitoring) facilitating behaviour change (Palsola et al., 2020). This could be one possible reason why changing social‐cognitive factors with an intervention in vocational schools is simply challenging to achieve.

In terms of shortcomings in the programme theory, the LMI developers had, for instance, reasons to assume that automatic, habitual influences play a role in behaviour, but had to exclude habit formation strategies for pragmatic reasons (Hankonen et al., 2020). Thus, the intervention might not have been effective enough to create new PA habits, leaving past (inactive) behaviours to overshadow everything else. Also, the programme theory did not tap into major environmental changes conducive to MVPA change. As LMI was found to increase (light) PA only during school hours (Hankonen et al., 2023), perhaps the trial's school‐time environmental changes (e.g. classroom activity breaks, standing desks) played a key role in inducing changes in light PA rather than the student component (e.g. classroom sessions, workbook and brochures). Thus, the programme theory for reducing SB might better tap into the changes in light PA (Aulbach et al., 2023) than the MVPA programme theory.

### Challenges of evaluating intervention programme theories and mechanisms of change

We followed Keele's (2015) recommendation to test change mechanisms separately due to problems in studying change mechanisms with mediation analysis (e.g. Bullock et al., 2010; Hofmann et al., 2020). The approach represents a current state‐of‐the‐art conceptualization of hypothesized mediating processes in behaviour change interventions (e.g. Hagger, Cameron, et al., 2020), but the caveats of the current practice of evaluating programme theories also pertain to the current study.

First, difficulties arise with high numbers of predictors. We presented a simplified model of a complex behaviour change intervention that includes multiple interacting components, targeting several behavioural factors on different levels of the individual's environment (Moore et al., 2015) which can also be described as a complex system (Shiell et al., 2008). Our model does not consider other known correlates of PA (e.g. automaticity, injunctive norm and controlled motivation), as they were not specified as key determinants and the intervention did not include components targeting these (see Hankonen et al., 2020). Left out of the causal model, their effect on PA is captured in the other variables and causal interpretation is not fully warranted (Bullock et al., 2010). A question of how to best evaluate the ‘mechanisms’ of behaviour change interventions arises when the intervention targets only a limited number of known behavioural determinants (e.g. habit in the current study). There are no straightforward answers to whether all the known factors should be considered when establishing mechanisms of change, for example as confounding variables, or if some should be left out of the modelling as not all influences can be accounted for in the statistical models, given limited sample sizes (Westfall & Yarkoni, 2016)—especially when it is not possible to distinguish between determinants, colliders and confounders (Rohrer, 2018). These concepts do not have clear‐cut roles in complex systems where most things are causally connected (often bi‐directionally, varying in time), creating difficulties for conventional models (Rickles, 2009).

Second, our models do not capture nonlinear, non‐additive effects. In complex systems, small effects can cause large differences, and processes are usually not additive nor linear (Heino et al., 2021; Hilborn, 2004). This is in line with theoretical thinking of the concepts: few social psychologists would argue that one unit of self‐efficacy produces the same effect at the lower and the higher end of a scale, or that self‐efficacy would not interact with other psychological constructs. Yet, the traditional statistical paradigm forces us to make these assumptions. While many solutions have been proposed for taking nonlinearity into account in regression models (e.g. González et al., 2010) and mediation (e.g. Knafl et al., 2017), they are not commonly applied and their grounding in linear regression requires making assumptions which may be unreasonable for complex systems (Wallot & Kelty‐Stephen, 2018; for a discussion in the behaviour change context, see Heino et al., 2021).

Third, the analytical strategy assumes a generic process of change for the entire intervention group. Group or population‐level data do not allow to directly draw inferences to the individual level (Fisher et al., 2018; Molenaar, 2007), yet theoretical mechanisms are almost exclusively tested with statistics that are informative only on the population level (Kwasnicka et al., 2019). This applies to all ‘non‐ergodic’ phenomena where individual differences (i.e. when people are high on x, they are high in *y*) do not equal those of temporal within‐individual processes (when individual's *x* rises, their *y* rises)—including most psychological objects of study (Hamaker, 2012). For example, in a design like the current one, the analysis does not allow concluding that the average changes in a mediating variable would be responsible for changing an individual's PA, although this is often the interpretation (Hofmann et al., 2020). Thus, in universal interventions, it is not only challenging to produce solutions that fit all individuals or subgroups, but also to find ways to evaluate the effectiveness of the programme theories.

Fourth, we evaluated constructs from different theories in the regression model, reflecting calls to integrate constructs from alternative models and test more comprehensive models (e.g. Baranowski et al., 1998). This approach relies on partialling out variance and can be problematic when the constructs are not conceptually independent (Crutzen et al., 2017; Crutzen & Peters, 2021; Gordon, 1968). For example, although autonomous motivation (from SDT) and intention (from RAA) refer to different concepts, they share features. Hence, the results from our stepwise regression do not pertain to the originally formulated theoretical concepts.

These four points highlight issues with determining causality in complex social systems using approaches that were developed in less complex settings such as agriculture—and later, medicine (Fisher, 1992). If behaviour change is considered non‐ergodic, idiographic, dynamical process (Heino et al., 2021; van Geert, 2019), issues such as attractor dynamics can severely hinder conclusions drawn from only a few observations per individual (Gernigon et al., 2022; Heino et al., 2022).

### Strengths and limitations

Strengths include a randomized design in a real‐world setting, objectively measured target behaviour, a relatively large sample size rare in social psychological studies, and the systematic evaluation of an integrative programme theory at two follow‐up points. Furthermore, the hypothesized programme theory was transparently registered prior to accessing the data. Insufficiently pre‐tested and thus insensitive measures of the determinants may have resulted in ceiling effects. This might be partly due to the possible lack of correspondence between the subjective social cognitive and objective PA measures, as subjective perception of what is ‘vigorous’ might lead to different relationships between these measures for those with poor and those with good physical fitness because the sense of intensity relates to one's fitness level.

Furthermore, studies with adolescents and young adults often encounter challenges with participants forgetting to wear the devices, and ours is no exception. To address the potential issue of wear‐time affecting the results, we used the standard 7‐day data collection and 4‐day and 10‐h criteria for data in this study. Also, the majority of participants who used accelerometers did use them according to our instructions.

### Future directions

Detecting changes in the use of behaviour change techniques deserves more investigations in the future. What are the key self‐enactable BCTs that drive the change in PA, or do the most effective BCTs vary across individuals (cf. Hankonen, 2020)? Future research should carefully pilot and test the sensitivity of the social‐cognitive measures beforehand, to avoid ceiling effects in the determinant variables. In hindsight, to ensure completion of all steps, we would recommend allocating 1 year for the feasibility study, followed by further 6–9 months for process evaluation and refinement of both the intervention and its assessment tools, prior to the randomized controlled trial. Furthermore, designs that take into account the conceptual differences between the within‐ and between‐individual processes should be utilized more widely (Molenaar, 2007). In sum, there is still work to be done on how to best develop and evaluate programme theories of real‐world interventions (Hankonen & Hardeman, 2020).

## CONCLUSIONS

Group differences were found only for BCT use and only autonomous motivation was positively associated with PA change. Although intervention effects on the theorized mechanisms of change were not detected in the entire sample, the intervention worked for some participants (Knittle, 2019). This study addressed the current lack of high‐quality assessments on theorized determinants hindering the identification of effective intervention strategies. This study advances the process evaluation literature by introducing multiple possibilities for future intervention programme theory evaluation efforts.

## AUTHOR CONTRIBUTIONS

**Minttu Palsola:** Conceptualization; data curation; formal analysis; investigation; visualization; writing – original draft; writing – review and editing; methodology. **Vera Araújo‐Soares:** Conceptualization; methodology; writing – review and editing. **Wendy Hardeman:** Conceptualization; methodology. **Ari Haukkala:** Conceptualization; formal analysis; writing – review and editing. **Matti Toivo Juhani Heino:** Data curation; writing – review and editing; investigation. **Falko Sniehotta:** Conceptualization; writing – review and editing; methodology. **Reijo Sund:** Data curation; formal analysis; writing – review and editing. **Tommi Vasankari:** Writing – review and editing. **Nelli Hankonen:** Conceptualization; funding acquisition; investigation; methodology; project administration; supervision; writing – original draft; writing – review and editing.

## CONFLICT OF INTEREST STATEMENT

The authors report no conflict of interest.

## ETHICS STATEMENT

The ethical committee of the Hospital District of Helsinki and Uusimaa (367/13/03/03/2014) reviewed the study procedures. The study procedures have been performed in accordance with the ethical standards as laid down in the 1964 Declaration of Helsinki and its later amendments or comparable ethical standards.

## Supporting information


Supplementary File 1.

Supplementary File 2.

## Data Availability

The data that support the findings of this study are available in the Finnish Social Science Data Archive at http://urn.fi/urn:nbn:fi:fsd:T‐FSD3446, reference number FSD3446.
