# Supplementary material for: Evaluating the Let's Move It intervention programme theory for adolescents' physical activity: Theorized psychosocial mechanisms of behavioural changes
Source: Br J Health Psychol. 2024 Sep 24;30(1):e12744. doi: 10.1111/bjhp.12744 (PMC11586702; doi:10.1111/bjhp.12744)
Supplement: Supplementary file 1 [file BJHP-30-0-s001.docx]

**Suppelementary file 1.** Intervention’s programme theory summarised, described as key theorized mediators linked to intervention content (Kostamo et al., 2019). See full description in Hankonen et al., (2020).

| **Theoretical determinant** | **Examples of BCTs linked to determinant** | **Examples of intervention content** |
| --- | --- | --- |
| Environmental resources and opportunities | In school:   - Environmental changes in classroom (physical equipment) (BCT 12.1, 12.5) - Teacher activity in classroom (e.g. activity breaks) (BCT 12.1; 12.2) - Better access to school PA facilities (BCT 12.1)   At home and in the neighbourhood:   - Home workout videos (BCT 12.1; 12.5) - Better access to neighbourhood PA opportunities  (BCT 12.1) - Social support | - *PA equipment, e.g. gym balls, standing desks, gym sticks, Pilates cushions provided in the classrooms* - *Teacher workshops, teacher guide, website* - *Improved access or improved awareness informed via leaflets in intervention sessions* - *Provision of home workout videos* - *Improved awareness of existing opportunities at home, online, environment* - *Arrangement of low-cost PA deals with community PA providers (informed via leaflets in student sessions)* |
| Positive outcome expectations  (both reflective and experiential) | - Information about health consequences (BCT 5.1); social and environmental consequences (BCT 5.3), and emotional consequences (BCT 5.6) - Behavioural experiments (BCT 4.4) | - *Student session activities: Focus on communicating positively framed benefits of increased activity, behavioural experiments (also as homework)* - *Workbook: Highlighted benefits of physical activity, exercises to find personally important benefits* - *Posters and brochures around the school to remind about the LMI principles and content, e.g., ”Any activity is better than nothing”, “Sitting sucks”.* |
| Autonomous motivation (intrinsic, integrated, identified motivation) | - Behavioural experiments (BCT 4.4) - Identification of oneself as active (self-concept); Identity associated with changed behaviour (BCT 13.5) | *Across sessions and materials:*   - *Autonomy supportive style* - *Principle “It is your choice”* - *Emphasis on selecting personally important reasons (principle “Know what moves you”)* - *Emphasis on selecting autonomous goals (incl. principle “Not fatless body but wellbeing”)* |
| Descriptive norms | - Information about others’ behaviour and attitudes toward PA (incl. information about others’ approval, BCT 6.3) | - *Student sessions: PA-related peer discussions and group activities* - *Workbook: Activities to engage in PA with friends* |
| Self-efficacy | - Verbal persuasion of capability (BCT 15.1) - Skill provision, Instruction on how to perform a behaviour (BCT 4.1) - Graded tasks (BCT 8.7) - Modelling, Demonstration of the behaviour (BCT 4.1) | - *Student sessions: Emphasis on principle ”Adding any activity is good”, focus on encouraging stories* - *Workbook: PA instructions, programs and apps, activities to find opportunities for incidental PA* - *Posters: Tips on ways to make days more active and how small changes matter* - *Brochures: Ways to identify and tackle barriers for PA* |
| Behavioural self-regulation | - Goal setting (behaviour) (BCT 1.1),  goal review (BCT 1.5), Discrepancy between current  behaviour and goal (BCT 1.6) - Action planning (BCT 1.4), coping planning (problem solving) (BCT 1.2) - Self-monitoring of behaviour (BCT 2.3) | - *Student session activities: Setting SMART goals and making PA plans, problem solving tasks related to PA barriers* - *Workbook: SMART planning template, physical activity diary template, checklists* - *Brochures: Tips and instructions on goal setting and monitoring, and on increasing incidental PA* |
| Intention | All activities directed at the other determinants were hypothesised to increase intention | *All the above.* |
