# Supplementary material for: Evaluating the Let's Move It intervention programme theory for adolescents' physical activity: Theorized psychosocial mechanisms of behavioural changes
Source: Br J Health Psychol. 2024 Sep 24;30(1):e12744. doi: 10.1111/bjhp.12744 (PMC11586702; doi:10.1111/bjhp.12744)
Supplement: Supplementary file 2 [file BJHP-30-0-s002.docx]

**Supplementary table 1.** Correlations between baseline psychological constructs and light physical activity (LPA) and moderate-to-vigorous physical activity (MVPA) at different measurement points.

|  | **BASELINE (T1)** | | | | | | | | | |  | **POST-INTERVENTION (T3)** | | | | | | | | | |  | **14-MONTH FOLLOW-UP** | | | | | | | | | |
| --- | --- | --- | --- | --- | --- | --- | --- | --- | --- | --- | --- | --- | --- | --- | --- | --- | --- | --- | --- | --- | --- | --- | --- | --- | --- | --- | --- | --- | --- | --- | --- | --- |
|  | **All days of the week (n=703-704)** | |  | **Weekdays  (n=695-696)** | |  | **Weekend (n=626-627)** | |  | **Self-reported  (n=1035-1038)** |  | **All days of the week (n=490-491)** | |  | **Weekdays  (n=481-482)** | |  | **Weekend (n=433-434)** | |  | **Self-reported  (n=845-847)** |  | **All days of the week (n=383-384)** | |  | **Weekdays  (n=381-382)** | |  | **Weekend (n=340-341)** | |  | **Self-reported  (n=635-637)** |
|  | **LPA** | **MVPA** |  | **LPA** | **MVPA** |  | **LPA** | **MVPA** |  | **Days of MVPA >30 mins for past 7 days)** |  | **LPA** | **MVPA** |  | **LPA** | **MVPA** |  | **LPA** | **MVPA** |  | **Days of MVPA >30 mins for past 7 days)** |  | **LPA** | **MVPA** |  | **LPA** | **MVPA** |  | **LPA** | **MVPA** |  | **Days of MVPA >30 mins for past 7 days)** |
| **BASELINE (T1)** |  |  |  |  |  |  |  |  |  |  |  |  |  |  |  |  |  |  |  |  |  |  |  |  |  |  |  |  |  |  |  |  |
| Opportunities | 0,033 | 0,048 |  | 0,031 | 0,043 |  | 0,046 | 0,046 |  | 0.181^**^ |  | 0.111^*^ | 0.132^**^ |  | 0.110^*^ | 0.120^**^ |  | 0,079 | 0.109^*^ |  | 0.157^**^ |  | 0,070 | 0,084 |  | 0,056 | 0,099 |  | 0,093 | 0,009 |  | 0.150^**^ |
| Positive outcome expectations | 0.165** | 0,026 |  | 0.151^**^ | 0,016 |  | 0.154^**^ | 0,056 |  | 0.138^**^ |  | 0.194^**^ | 0.105^*^ |  | 0.167^**^ | 0.110^*^ |  | 0.189^**^ | 0,084 |  | 0.148^**^ |  | 0.248^**^ | 0,076 |  | 0.254^**^ | 0,080 |  | 0.190^**^ | 0,073 |  | 0,071 |
| Autonomous motivation | 0.236^**^ | 0.245^**^ |  | 0.233^**^ | 0.231^**^ |  | 0.180^**^ | 0.222^**^ |  | 0.461^**^ |  | 0.278^**^ | 0.318^**^ |  | 0.262^**^ | 0.307^**^ |  | 0.260^**^ | 0.263^**^ |  | 0.415^**^ |  | 0.325^**^ | 0.299^**^ |  | 0.302^**^ | 0.277^**^ |  | 0.261^**^ | 0.240^**^ |  | 0.315^**^ |
| Descriptive norm | 0.115^**^ | 0,060 |  | 0.107^**^ | 0,046 |  | 0.105^**^ | 0,059 |  | 0.253^**^ |  | 0.161^**^ | 0.114^*^ |  | 0.165^**^ | 0.123^**^ |  | 0.126^**^ | 0,086 |  | 0.210^**^ |  | 0.156^**^ | 0.146^**^ |  | 0.131^*^ | 0.143^**^ |  | 0.163^**^ | 0.107^*^ |  | 0.143^**^ |
| Self-efficacy / perceived behavioural control | 0,011 | 0.109^**^ |  | -0,006 | 0.088^*^ |  | 0,061 | 0.107^**^ |  | 0.204^**^ |  | 0,037 | 0.096^*^ |  | 0,062 | 0,087 |  | -0,032 | 0,079 |  | 0.217^**^ |  | 0,082 | 0.130^*^ |  | 0,074 | 0.122^*^ |  | 0,046 | 0,044 |  | 0.149^**^ |
| Intention | 0.228^**^ | 0.178^**^ |  | 0.217^**^ | 0.152^**^ |  | 0.206^**^ | 0.180^**^ |  | 0.376^**^ |  | 0.277^**^ | 0.221^**^ |  | 0.269^**^ | 0.208^**^ |  | 0.231^**^ | 0.173^**^ |  | 0.375^**^ |  | 0.311^**^ | 0.250^**^ |  | 0.275^**^ | 0.220^**^ |  | 0.290^**^ | 0.195^**^ |  | 0.240^**^ |
| BCT use (identification of use) | 0.188^**^ | 0.176^**^ |  | 0.195^**^ | 0.176^**^ |  | 0.123^**^ | 0.117^**^ |  | 0.426^**^ |  | 0.210^**^ | 0.227^**^ |  | 0.198^**^ | 0.232^**^ |  | 0.155^**^ | 0.142^**^ |  | 0.414^**^ |  | 0.354^**^ | 0.248^**^ |  | 0.320^**^ | 0.221^**^ |  | 0.319^**^ | 0.220^**^ |  | 0.309^**^ |
| BCT use (frequency of use) | 0.165^**^ | 0.145^**^ |  | 0.172^**^ | 0.153^**^ |  | 0.105^**^ | 0.080^*^ |  | 0.380^**^ |  | 0.186^**^ | 0.206^**^ |  | 0.170^**^ | 0.203^**^ |  | 0.144^**^ | 0.120^*^ |  | 0.357^**^ |  | 0.323^**^ | 0.191^**^ |  | 0.279^**^ | 0.177^**^ |  | 0.301^**^ | 0.134^*^ |  | 0.251^**^ |
| Action planning | 0.162^**^ | 0.144^**^ |  | 0.159^**^ | 0.140^**^ |  | 0.102^*^ | 0.113^**^ |  | 0.351^**^ |  | 0.235^**^ | 0.228^**^ |  | 0.238^**^ | 0.218^**^ |  | 0.202^**^ | 0.194^**^ |  | 0.371^**^ |  | 0.253^**^ | 0.221^**^ |  | 0.230^**^ | 0.186^**^ |  | 0.239^**^ | 0.207^**^ |  | 0.272^**^ |
| Coping planning | 0.155^**^ | 0.164^**^ |  | 0.145^**^ | 0.171^**^ |  | 0.133^**^ | 0.116^**^ |  | 0.338^**^ |  | 0.184^**^ | 0.235^**^ |  | 0.190^**^ | 0.222^**^ |  | 0.128^**^ | 0.205^**^ |  | 0.359^**^ |  | 0.240^**^ | 0.202^**^ |  | 0.229^**^ | 0.184^**^ |  | 0.216^**^ | 0.160^**^ |  | 0.287^**^ |

**Correlation is significant at the 0.01 level (2-tailed).
*Correlation is significant at the 0.05 level (2-tailed).

**Supplementary table 2.** Correlations between post-intervention psychological constructs and light physical activity (LPA) and moderate-to-vigorous physical activity (MVPA) at different measurement points.

|  | **BASELINE (T1)** | | | | | | | | | | |  | | **POST-INTERVENTION (T3)** | | | | | | | | | |  | **14-MONTH FOLLOW-UP** | | | | | | | | | |
| --- | --- | --- | --- | --- | --- | --- | --- | --- | --- | --- | --- | --- | --- | --- | --- | --- | --- | --- | --- | --- | --- | --- | --- | --- | --- | --- | --- | --- | --- | --- | --- | --- | --- | --- |
|  | **All days of the week (n=622-625)** | |  | **Weekdays  (n=615-618)** | |  | **Weekend (n=552-554)** | |  | **Self-reported  (n=838-844)** |  | | **All days of the week (n=480-483)** | | |  | **Weekdays  (n=471-474)** | |  | **Weekend (n=425-427)** | |  | **Self-reported  (n=893-898)** |  | **All days of the week (n=362-364)** | |  | **Weekdays  (n=360-362)** | |  | **Weekend (n=317-320)** | |  | **Self-reported  (n=576-580)** |
|  | **LPA** | **MVPA** |  | **LPA** | **MVPA** |  | **LPA** | **MVPA** |  | **Days of MVPA >30 mins for past 7 days)** |  | | **LPA** | | **MVPA** |  | **LPA** | **MVPA** |  | **LPA** | **MVPA** |  | **Days of MVPA >30 mins for past 7 days)** |  | **LPA** | **MVPA** |  | **LPA** | **MVPA** |  | **LPA** | **MVPA** |  | **Days of MVPA >30 mins for past 7 days)** |
| **POST-INTERVENTION (T3)** |  |  |  |  |  |  |  |  |  |  |  | |  | |  |  |  |  |  |  |  |  |  |  |  |  |  |  |  |  |  |  |  |  |
| Opportunities | 0,047 | 0,013 |  | 0,024 | 0,005 |  | 0,077 | -0,009 |  | 0.130^**^ |  | | 0,026 | | -0,002 |  | 0,042 | -0,027 |  | -0,015 | 0,005 |  | 0.112^**^ |  | 0,043 | 0,044 |  | 0,035 | 0,040 |  | 0,022 | 0,011 |  | 0.099^*^ |
| Positive outcome expectations | 0.161^**^ | 0,054 |  | 0.150^**^ | 0,050 |  | 0.126^**^ | 0,055 |  | 0.115^**^ |  | | 0.191^**^ | | 0,049 |  | 0.174^**^ | 0,065 |  | 0.152^**^ | -0,027 |  | 0.155^**^ |  | 0.287^**^ | 0.113^*^ |  | 0.262^**^ | 0,090 |  | 0.265^**^ | 0.121^*^ |  | 0.136^**^ |
| Autonomous motivation | 0.260^**^ | 0.238^**^ |  | 0.248^**^ | 0.224^**^ |  | 0.202^**^ | 0.204^**^ |  | 0.356^**^ |  | | 0.305^**^ | | 0.251^**^ |  | 0.286^**^ | 0.240^**^ |  | 0.243^**^ | 0.169^**^ |  | 0.407^**^ |  | 0.347^**^ | 0.220^**^ |  | 0.315^**^ | 0.185^**^ |  | 0.308^**^ | 0.213^**^ |  | 0.292^**^ |
| Descriptive norm | 0.085^*^ | 0,026 |  | 0,068 | 0,000 |  | 0.093^*^ | 0,058 |  | 0.216^**^ |  | | 0.103^*^ | | 0,056 |  | 0.095^*^ | 0,054 |  | 0.134^**^ | 0,052 |  | 0.254^**^ |  | 0,098 | 0.125^*^ |  | 0,083 | 0.131^*^ |  | 0.110^*^ | 0,101 |  | 0.197^**^ |
| Self-efficacy / perceived behavioural control | 0,030 | 0.109^**^ |  | 0,020 | 0.087^*^ |  | 0,038 | 0.109^*^ |  | 0.171^**^ |  | | 0,019 | | 0.107^*^ |  | 0,023 | 0.093^*^ |  | 0,013 | 0,090 |  | 0.228^**^ |  | 0,094 | 0.109^*^ |  | 0,092 | 0,086 |  | 0,028 | 0.138^*^ |  | 0.154^**^ |
| Intention | 0.221^**^ | 0.139^**^ |  | 0.221^**^ | 0.131^**^ |  | 0.160^**^ | 0.123^**^ |  | 0.324^**^ |  | | 0.228^**^ | | 0.193^**^ |  | 0.240^**^ | 0.189^**^ |  | 0.120^*^ | 0.140^**^ |  | 0.414^**^ |  | 0.260^**^ | 0.202^**^ |  | 0.264^**^ | 0.186^**^ |  | 0.180^**^ | 0.153^**^ |  | 0.227^**^ |
| BCT use (identification of use) | 0.222^**^ | 0.200^**^ |  | 0.204^**^ | 0.192^**^ |  | 0.193^**^ | 0.171^**^ |  | 0.334^**^ |  | | 0.232^**^ | | 0.164^**^ |  | 0.222^**^ | 0.176^**^ |  | 0.161^**^ | 0,087 |  | 0.449^**^ |  | 0.259^**^ | 0.124^*^ |  | 0.258^**^ | 0.122^*^ |  | 0.200^**^ | 0,104 |  | 0.282^**^ |
| BCT use (frequency of use) | 0.193^**^ | 0.149^**^ |  | 0.179^**^ | 0.147^**^ |  | 0.162^**^ | 0.127^**^ |  | 0.291^**^ |  | | 0.238^**^ | | 0.156^**^ |  | 0.214^**^ | 0.140^**^ |  | 0.212^**^ | 0.116^*^ |  | 0.365^**^ |  | 0.228^**^ | 0.109^*^ |  | 0.225^**^ | 0.115^*^ |  | 0.195^**^ | 0,061 |  | 0.203^**^ |
| Action planning | 0.204^**^ | 0.126^**^ |  | 0.189^**^ | 0.116^**^ |  | 0.183^**^ | 0.108^*^ |  | 0.262^**^ |  | | 0.231^**^ | | 0.141^**^ |  | 0.225^**^ | 0.140^**^ |  | 0.170^**^ | 0,057 |  | 0.420^**^ |  | 0.223^**^ | 0.170^**^ |  | 0.201^**^ | 0.155^**^ |  | 0.197^**^ | 0.162^**^ |  | 0.302^**^ |
| Coping planning | 0.170^**^ | 0.140^**^ |  | 0.141^**^ | 0.131^**^ |  | 0.160^**^ | 0.106^*^ |  | 0.302^**^ |  | | 0.180^**^ | | 0.139^**^ |  | 0.164^**^ | 0.154^**^ |  | 0.133^**^ | 0,028 |  | 0.418^**^ |  | 0.224^**^ | 0.140^**^ |  | 0.214^**^ | 0.129^*^ |  | 0.192^**^ | 0.143^*^ |  | 0.302^**^ |

**Correlation is significant at the 0.01 level (2-tailed).
*Correlation is significant at the 0.05 level (2-tailed).

**Supplementary table 3.** Correlations between follow-up psychological constructs and light physical activity (LPA) and moderate-to-vigorous physical activity (MVPA) at different measurement points.

|  | **BASELINE (T1)** | | | | | | | | | | |  | | **POST-INTERVENTION (T3)** | | | | | | | | | |  | **14-MONTH FOLLOW-UP** | | | | | | | | | |
| --- | --- | --- | --- | --- | --- | --- | --- | --- | --- | --- | --- | --- | --- | --- | --- | --- | --- | --- | --- | --- | --- | --- | --- | --- | --- | --- | --- | --- | --- | --- | --- | --- | --- | --- |
|  | **All days of the week (n=467-470)** | |  | **Weekdays  (n=462-467)** | |  | **Weekend (n=414-417)** | |  | **Self-reported  (n=627-635)** |  | | **All days of the week (n=346-349)** | | |  | **Weekdays  (n=341-343)** | |  | **Weekend (n=313-316)** | |  | **Self-reported  (n=572-580)** |  | **All days of the week (n=381-385)** | |  | **Weekdays  (n=379-383)** | |  | **Weekend (n=342-345)** | |  | **Self-reported  (n=648-657)** |
|  | **LPA** | **MVPA** |  | **LPA** | **MVPA** |  | **LPA** | **MVPA** |  | **Days of MVPA >30 mins for past 7 days)** |  | | **LPA** | | **MVPA** |  | **LPA** | **MVPA** |  | **LPA** | **MVPA** |  | **Days of MVPA >30 mins for past 7 days)** |  | **LPA** | **MVPA** |  | **LPA** | **MVPA** |  | **LPA** | **MVPA** |  | **Days of MVPA >30 mins for past 7 days)** |
| **14-MONTH FOLLOW-UP (T4)** |  |  |  |  |  |  |  |  |  |  |  | |  | |  |  |  |  |  |  |  |  |  |  |  |  |  |  |  |  |  |  |  |  |
| Opportunities | 0,008 | 0,044 |  | 0,021 | 0,035 |  | 0,007 | 0,037 |  | 0.086^*^ |  | | 0,063 | | 0,033 |  | 0,099 | 0,007 |  | -0,016 | 0,062 |  | 0,036 |  | 0,055 | 0,070 |  | 0,063 | 0,056 |  | -0,004 | 0,025 |  | 0.104^**^ |
| Positive outcome expectations | 0.159^**^ | 0,047 |  | 0.152^**^ | 0,021 |  | 0.181^**^ | 0,089 |  | 0,050 |  | | 0.225^**^ | | 0,060 |  | 0.253^**^ | 0,042 |  | 0,071 | 0,060 |  | 0.088^*^ |  | 0.275^**^ | 0,092 |  | 0.269^**^ | 0,071 |  | 0.198^**^ | 0,085 |  | 0,069 |
| Autonomous motivation | 0.224^**^ | 0.253^**^ |  | 0.219^**^ | 0.238^**^ |  | 0.201^**^ | 0.215^**^ |  | 0.315^**^ |  | | 0.340^**^ | | 0.283^**^ |  | 0.351^**^ | 0.239^**^ |  | 0.233^**^ | 0.278^**^ |  | 0.360^**^ |  | 0.298^**^ | 0.292^**^ |  | 0.279^**^ | 0.272^**^ |  | 0.257^**^ | 0.220^**^ |  | 0.375^**^ |
| Descriptive norm | 0.118^*^ | 0.105^*^ |  | 0.134^**^ | 0,090 |  | 0,077 | 0,074 |  | 0.235^**^ |  | | 0.156^**^ | | 0.139^**^ |  | 0.167^**^ | 0.116^*^ |  | 0.120^*^ | 0.178^**^ |  | 0.217^**^ |  | 0.138^**^ | 0.136^**^ |  | 0.137^**^ | 0.115^*^ |  | 0.111^*^ | 0.143^**^ |  | 0.192^**^ |
| Self-efficacy / perceived behavioural control | -0,066 | 0.092^*^ |  | -0,061 | 0,066 |  | -0,032 | 0.108^*^ |  | 0.090^*^ |  | | 0,027 | | 0,073 |  | 0,061 | 0,043 |  | -0,042 | 0,106 |  | 0.083^*^ |  | 0,071 | 0.130^*^ |  | 0,084 | 0.117^*^ |  | 0,001 | 0,102 |  | 0.188^**^ |
| Intention | 0.210^**^ | 0.251^**^ |  | 0.182^**^ | 0.221^**^ |  | 0.224^**^ | 0.222^**^ |  | 0.327^**^ |  | | 0.280^**^ | | 0.267^**^ |  | 0.296^**^ | 0.243^**^ |  | 0.219^**^ | 0.212^**^ |  | 0.328^**^ |  | 0.229^**^ | 0.290^**^ |  | 0.225^**^ | 0.269^**^ |  | 0.174^**^ | 0.219^**^ |  | 0.438^**^ |
| BCT use (identification of use) | 0.193^**^ | 0.185^**^ |  | 0.205^**^ | 0.189^**^ |  | 0.160^**^ | 0.129^**^ |  | 0.315^**^ |  | | 0.269^**^ | | 0.242^**^ |  | 0.270^**^ | 0.211^**^ |  | 0.173^**^ | 0.214^**^ |  | 0.317^**^ |  | 0.239^**^ | 0.251^**^ |  | 0.227^**^ | 0.244^**^ |  | 0.202^**^ | 0.163^**^ |  | 0.385^**^ |
| BCT use (frequency of use) | 0.218^**^ | 0.193^**^ |  | 0.237^**^ | 0.200^**^ |  | 0.169^**^ | 0.100^*^ |  | 0.263^**^ |  | | 0.279^**^ | | 0.214^**^ |  | 0.285^**^ | 0.176^**^ |  | 0.220^**^ | 0.241^**^ |  | 0.284^**^ |  | 0.210^**^ | 0.223^**^ |  | 0.203^**^ | 0.216^**^ |  | 0.219^**^ | 0.147^**^ |  | 0.297^**^ |
| Action planning | 0.145^**^ | 0.211^**^ |  | 0.141^**^ | 0.183^**^ |  | 0.127^**^ | 0.184^**^ |  | 0.304^**^ |  | | 0.176^**^ | | 0.196^**^ |  | 0.195^**^ | 0.171^**^ |  | 0.146^**^ | 0.178^**^ |  | 0.278^**^ |  | 0.181^**^ | 0.233^**^ |  | 0.167^**^ | 0.224^**^ |  | 0.149^**^ | 0.165^**^ |  | 0.425^**^ |
| Coping planning | 0.141^**^ | 0.194^**^ |  | 0.137^**^ | 0.177^**^ |  | 0.122^*^ | 0.146^**^ |  | 0.336^**^ |  | | 0.184^**^ | | 0.248^**^ |  | 0.199^**^ | 0.238^**^ |  | 0.113^*^ | 0.179^**^ |  | 0.326^**^ |  | 0.164^**^ | 0.231^**^ |  | 0.167^**^ | 0.231^**^ |  | 0.126^*^ | 0.162^**^ |  | 0.431^**^ |

**Correlation is significant at the 0.01 level (2-tailed).
*Correlation is significant at the 0.05 level (2-tailed).
